# Clinical Prediction Models for Contact X‐Ray Brachytherapy in Managing Rectal Cancers: A Scoping Review

**DOI:** 10.1002/cam4.70697

**Published:** 2025-04-03

**Authors:** Muneeb Ul Haq, D. Mark Pritchard, Arthur Sun Myint, Muhammad Ahsan Javed, Carrie A. Duckworth, Ngu Wah Than, Laura J. Bonnett, David M. Hughes

**Affiliations:** ^1^ Institute of Systems, Molecular and Integrative Biology The University of Liverpool Liverpool UK; ^2^ The Clatterbridge Cancer Centre NHS Foundation Trust Liverpool UK; ^3^ Liverpool University Hospitals NHS Foundation Trust Liverpool UK; ^4^ Institute of Life Course and Medical Sciences The University of Liverpool Liverpool UK; ^5^ Department of Health Data Science, Institute of Population Health The University of Liverpool Liverpool UK

**Keywords:** brachytherapy, clinical prediction models, rectal cancers

## Abstract

**Background:**

Currently, there are no clinically predictive models that can prognosticate the response of rectal cancers to Contact X‐ray brachytherapy (CXB). This review aims to critically evaluate existing models that have attempted to predict the response of rectal cancer to external beam radiotherapy, with the objective of laying the foundation for the development of a CXB‐specific prediction model.

**Methods:**

A random‐effects meta‐analysis was employed to calculate pooled estimates of the discriminative ability of published models. Using the Prediction Model Risk Of Bias Assessment Tool (PROBAST), each model was evaluated for its risk of bias and applicability. Additionally, the frequency of commonly utilised predictive factors was documented.

**Results:**

Twelve papers discussed fifteen models based on pre‐treatment factors. Models predicting response based on the Tumour regression grade (TRG) classified responders as patients who achieved a complete response or near complete response and achieved a pooled AUC of 0.82 (95% CI 0.74–0.89). Models that predicted pathologic complete response (pCR) had a pooled AUC of 0.76 (95% CI 0.71–0.82). The most utilised predictive parameters were age, tumour grade and T stage. However, these models were prone to significant risk of bias and had limited applicability to the general population.

**Conclusions:**

Although the existing models were statistically robust, they lacked broad applicability. This was primarily due to a lack of external validation, which limits their clinical utility. A future CXB‐specific model should prioritise dedicated data collection based on pre‐calculated sample size and include the predictive factors identified in this review.

## 1 Introduction

Colorectal cancers rank third in global incidence and contribute to approximately one‐tenth of all cancer‐related mortalities [1]. Rectal cancers account for approximately one‐third of all colorectal cancer cases [2]. In the UK and many other countries, radical surgery, after neoadjuvant chemoradiotherapy or short‐course radiotherapy and consolidation chemotherapy, remains the standard of care for the treatment of locally advanced rectal cancer (LARC) [3]. However, elderly patients are often not ideal surgical candidates. This age group is associated with a higher risk of post‐operative morbidity and mortality [4].

Various national bowel‐screening programmes, such as the one in the UK, have lowered the age at which screening for bowel cancer begins. However, it is important to recognise that rectal cancer remains a disease that primarily affects the elderly [1]. In countries which have extended screening programs, such as Australia, the median age of diagnosed patients remains at 65. This trend highlights that a significant proportion of patients diagnosed is still likely to be elderly and therefore less suitable candidates for surgery [2].

In cases where rectal cancer patients attain a clinical complete response (cCR) after external beam (chemo)radiation (EBCRT), active monitoring of tumour recurrence becomes a viable alternative option to surgery [5]. This watch‐and‐wait protocol preserves anal sphincter function while also avoiding potential surgical complications. However, approximately a third of patients develop local tumour regrowth. These patients then need salvage surgery for definitive management [6]. One possible strategy to reduce the incidence of local tumour regrowth involves giving a higher dosage of external beam radiation therapy (54 Gy). However, this comes at the cost of an increased likelihood of side effects. Therefore, a more favourable option, which appears to significantly reduce local recurrence rates and which is associated with fewer side effects, is the implementation of a contact X‐ray brachytherapy boost to the EBRT regimen [7, 8].

Contact X‐ray brachytherapy (CXB), also known as Papillon, is a high‐dose low‐energy radiation that has the potential to preserve anal sphincter function in locally advanced rectal cancer patients without compromising their oncological outcomes [9]. In early rectal tumours < 3 cm in diameter, it can be used in de‐novo patients as a standalone radical treatment. For larger tumours, it can be used as a boost following external beam radiotherapy (EBRT), and for increasing local control after local tumour excision when high‐risk features are found on pathology [10, 11]. The UK National Institute for Health and Care Excellence (NICE) has recommended this treatment for rectal cancer patients who are not suitable for surgery [12]. Organ Preservation for Early Rectal Adenocarcinoma (OPERA), a phase 3 randomised controlled clinical trial, has recently shown a significantly higher 3‐year organ preservation rate of more than 80% for tumours 3 cm or larger and a 97% organ preservation rate for tumours measuring < 3 cm, in a cohort of patients who were eligible candidates for surgery, using CXB in addition to external beam chemoradiotherapy [13].

Despite these findings, surgery following neoadjuvant treatment remains the established standard of care for LARC in the UK and many other countries. Therefore, to improve the effectiveness of neoadjuvant treatments such as CXB or EBRT and to provide non‐operative management options to patients, it is important to optimise patient selection.

Specific factors such as administering CXB before EBRT for tumours < 3 cm in diameter and the magnitude of the initial clinical response to CXB predict a favourable prognosis in CXB‐treated patients [13, 14]. In contrast, various patient‐related features such as performance status, age at diagnosis, distance from the anal verge, tumour size, T stage and N stage at presentation lack statistical significance in predicting initial treatment response or local regrowth rate in published series. However, disease‐free survival has been shown to correlate with performance status, age and the radiotherapy regime used [15].

A clinical prediction model is a combination of prognostic factors (clinical variables) which, when combined within a mathematical model, can predict an individual's treatment outcome. Clinical prediction models can be used to identify patients who are more likely to have a more favourable outcome after receiving a treatment such as CXB [16].

Currently, there are no clinically useful clinical prediction models for rectal cancer patients who are undergoing CXB treatment. Therefore, our aim was to systematically identify, summarise and critically appraise published prediction models that have evaluated the responses of radiotherapy in rectal adenocarcinomas. This review focused on models that relied solely on pre‐treatment predictive factors. The objective of this review is to contribute valuable insights to guide the development of future prediction models designed to predict the response of rectal cancer patients to CXB.

## 2 Methods

This review was performed in accordance with the Preferred Reporting Items for Systematic Review and Meta‐analyses (PRISMA) guidelines [17]. The protocol for this review was prospectively registered on the International prospective register of systematic reviews (PROSPERO) (CRD42022277704) [18]. An important amendment to this protocol was that Muneeb Ul Haq (MH) replaced Margarita Karageorgou (MK) as the primary reviewer.

### 2.1 Literature Search

PubMed, Embase, Scopus, Web of Science, Cochrane CENTRAL and Medical Literature Analysis and Retrieval System Online (MEDLINE) databases were examined. Haynes broad filter is specifically designed for differentiating papers reporting on prediction models from predictive factors [19]. This was used in combination with an additional search string developed by combining search terms “Rectal Cancer” “Contact Brachytherapy” “Radiotherapy” and their synonym terminologies. The search string utilised is shown in Table S1. This combination has the potential to increase the sensitivity of our search string up to 0.9–1.0 [19].

Title and abstract screening were performed by the primary reviewer (MH), with the second reviewer (DH) independently assessing 10% of the total articles (at each stage). To ensure transparency, the research team utilised a web‐based application, Rayyan [20]. All reviewers were initially blinded to the decisions of their peers, and the blinding was only lifted for the purposes of resolving disputes. Disagreements between reviewers were resolved by discussion, and all persisting conflicts were resolved by discussion with a third reviewer (LB).

### 2.2 Eligibility Criteria

The eligibility criteria were refined based on the initial search results. Rectal cancers were defined as adenocarcinomas and their subtypes, such as mucinous rectal adenocarcinomas. Other histological types of rectal neoplasm were excluded. Similarly, a prediction tool/model was defined as a combination of two or more factors that could predict patients' responses to radiotherapy. If predictive factors were amalgamated into a composite score, studies were only included when the assigned weights were proportionate to a factor's regression coefficient. Models that indiscriminately assigned equal weights to all included factors were not included.

Studies reporting the extension of a previously published model, that is, reporting on the effect of incrementally adding new predictors to published models, were excluded. Similarly, studies that only measured an existing model's predictive accuracy in an independent population (compared to the model's training cohort), that is, validation studies, were excluded. Studies that revised an existing model by performing slope and intercept recalibration and refitting of one or more coefficients were treated similarly. In the above cases, the original model was manually retrieved and included in the review.

Our objective was to identify models predicting response to radiotherapy at baseline. Therefore, utilisation of post‐treatment factors, such as post‐radiation T stage, led to exclusion from this review.

Similarly, models based on radiomics were also excluded as they are out of the scope of this review. This is because the Cochrane review group has yet to publish guidelines for evaluating the risk of bias in radiomic models. Furthermore, imaging alone has limited utility in predicting treatment outcomes.

### 2.3 Data Extraction

Only original articles published from the point of inception of the database up until August 2023 were considered. Data extraction was done using the Critical Appraisal and Data Extraction for Systematic Reviews of Prediction Modelling Studies (CHARMS) checklist. If an article described multiple models, separate data extraction was carried out for each model.

All papers included in our review underwent evaluation using the Prediction Model Risk of Bias Assessment Tool (PROBAST) to evaluate the risk of bias in model development and the applicability of the proposed predictive model [21]. According to PROBAST, a high risk of bias in any single domain predisposes the model to have an overall high bias.

The risk‐of‐bias and applicability assessment was carried out by two independent investigators. Domains such as participants, predictors, outcomes and analysis were evaluated for the risk of bias. Whereas for evaluating the applicability of the participants, predictors and outcomes were examined [21].

### 2.4 Statistical and Descriptive Analyses

In this review, there was a wide range of effect sizes across different models. We conducted a random‐effects model meta‐analyses by pooling data from models assessing similar outcomes, such as clinical complete response or pathological complete response.

For comparison of calibration measures, we treated the C‐statistic and the area under the curve (AUC) interchangeably, particularly when evaluating models with binary outcomes, that is, pathological complete response (pCR or no pCR) [22]. Similarly, patients were defined as responders based on the Tumour regression grade (TRG) if they achieved a complete response or near complete response after neoadjuvant therapy. To estimate the summary effect size and its 95% confidence interval, we employed random‐effects models based on the DerSimonian and Laird (DL) method to account for the heterogeneity in terms of sample size between studies [23]. Since the AUC is non‐parametric, we utilised DeLong's method to convert the variance into a 95% confidence interval [22].

Meta‐analyses were undertaken using the ‘rma()’ function from the ‘metafor’ package using the statistical software R [24]. Finally, the *I* ^2^ statistic was calculated to measure the percentage of total variability due to between‐study heterogeneity.

## 3 Results

Our search strategy identified 5933 studies (after removing duplicates). Following title screening, 5692 publications were excluded. Additionally, 220 studies were excluded during abstract screening.

**FIGURE 1:**
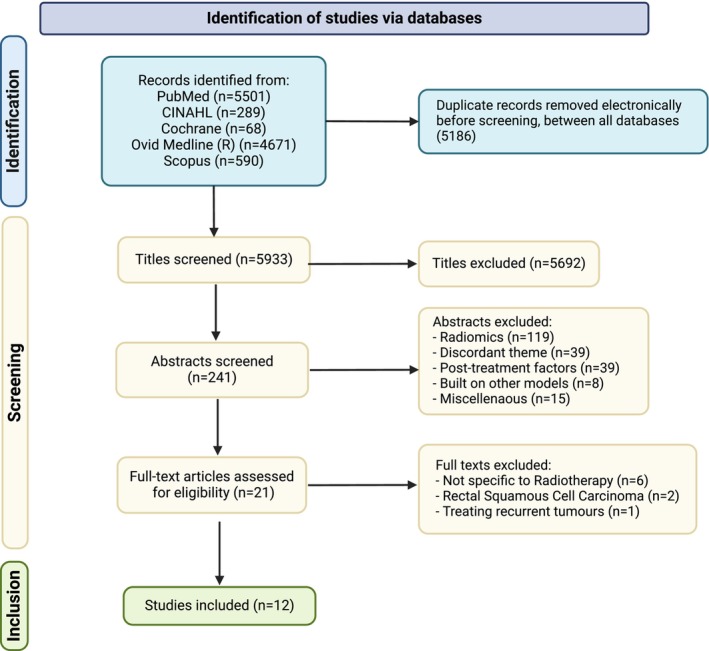
PRISMA flowchart representing the study screening and selection process.

These exclusions were primarily due to the use of post‐treatment factors in model development or due to radiomics‐based models. Subsequently, 21 articles were considered for full‐text screening. Six of these papers, which described non‐radiotherapy‐specific models, two papers that were focused on squamous cell carcinomas, and one paper that reported on recurrent tumours were subsequently excluded. The PRISMA Flow chart is shown in Figure 1.

Finally, 12 papers, all published before April 2023, met our predefined inclusion and exclusion criteria [25, 26, 27, 28, 29, 30, 31, 32, 33, 34, 35, 36]. 15 individual prediction models were extracted from these 12 publications. Despite significant heterogeneity between models based on sample size, radiotherapy dose standardisation and the completeness of model reporting (discussed later), we performed a meta‐analysis to evaluate the predictive performance of these models.

**FIGURE 2:**
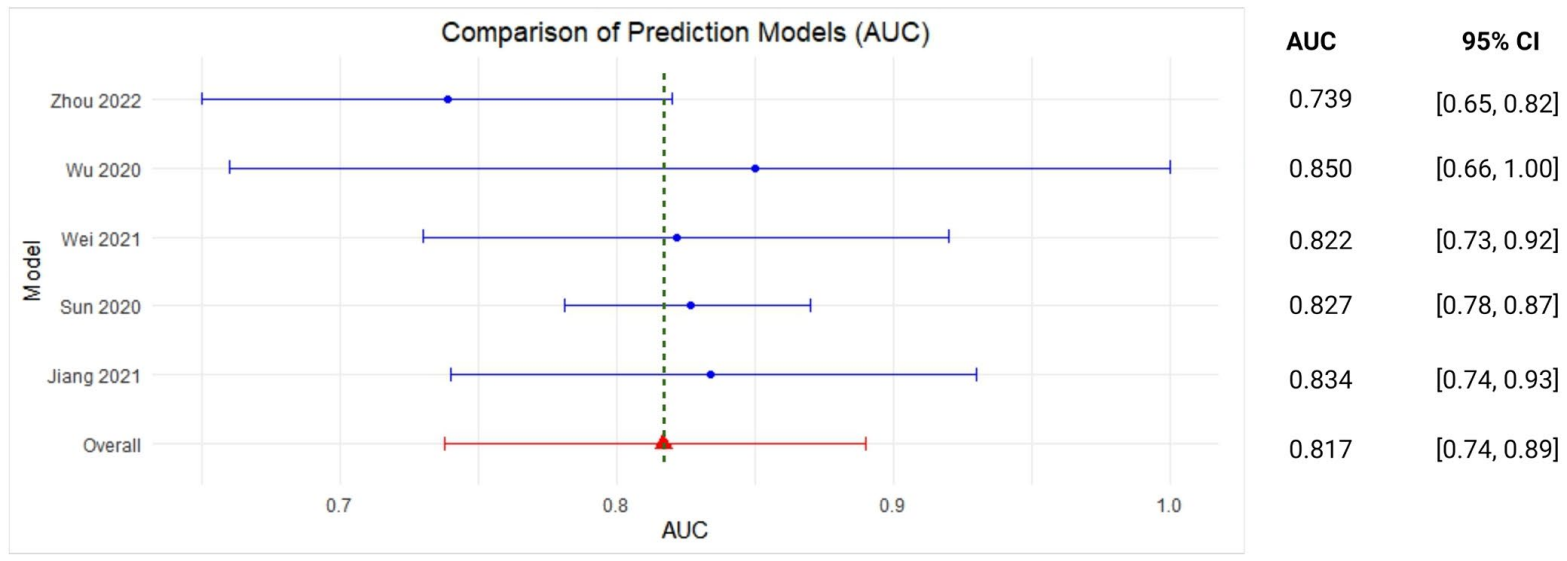
Meta‐analysis forest plot for clinical prediction models predicting response based on Tumour Regression Grade (TRG).

**FIGURE 3:**
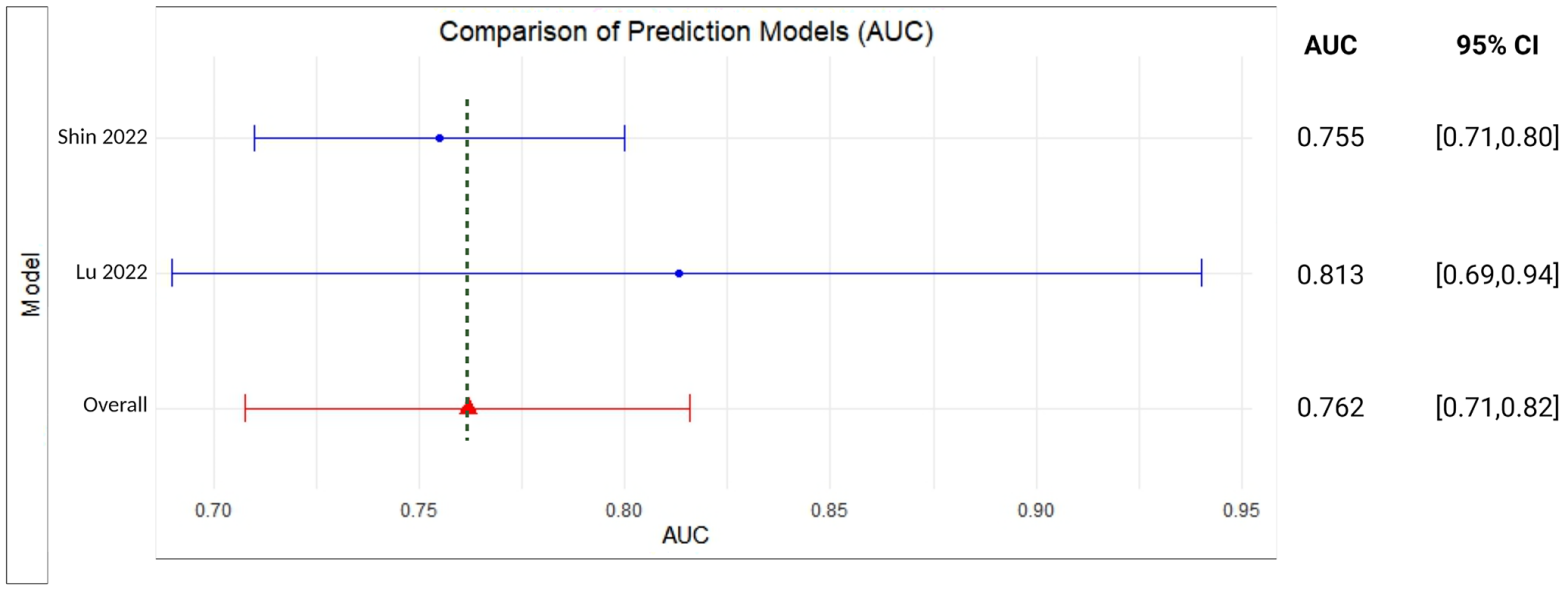
Meta‐analysis forest plot for clinical prediction models predicting response based on pathological response (pCR).

A subset of these models predicted rates of histopathological response based on Tumour Regression Grade (TRG) [25, 29, 30, 32, 33] Area Under the Curve (AUC) of models evaluating TRG was 0.82 (95% CI 0.74–0.89) with an *I* ^2^ value of 10.66%. For rates of pathological complete response (pCR) [31, 35], the overall reported AUC was 0.76 (95% CI: 0.71–0.82) with an *I* ^2^ value of 10.97%. These results are represented in Figures 2 and 3, respectively. However, the results should be interpreted with caution and correlated with the additional features and limitations discussed in subsequent sections.

### 3.1 Study Characteristics

In total, 15 different models were extracted from the 12 individual studies included in our review. Most papers recruited patients from cancer hospitals alone (*n* = 8) [25, 26, 30, 31, 32, 33, 34, 35] or in combination with research labs (*n* = 2) [28, 36]. A good geographical diversity was observed, with China (*n* = 5) [25, 30, 31, 32, 33] and the USA (*n* = 2) [28, 34] being the major contributors. Canada, South Korea and the UK contributed to one paper each [26, 35, 36] while Italy and Japan contributed to a proportion of patients alongside the USA [28]. Two papers did not specify the geographical background of the recruited population [27, 29].

Retrospective data collection was the prevalent method, and four studies had dedicated data collection for the purpose of building their models [30, 31, 33, 35]. Five papers reported using pre‐existing registries [27, 28, 29, 34, 36]. Only three studies attempted to prospectively recruit patients [25, 26, 32]. All of these were single‐centre studies.

The included studies reported a male to female ratio of 60:40. This gender ratio is consistent with the current trends observed about the epidemiology of rectal cancers. Similarly, there was low variability between cohorts based on T staging, with some showing a prevalence of more advanced T stages (T3 [24, 30, 31, 32, 33, 34, 35, 36, 37], T4 [33]). These were based on different editions (6th to 8th) of the American Joint Committee on Cancer (AJCC) TNM staging criteria, with similar staging performance between all criteria [38].

The age distribution across studies was reported in various formats; two papers each utilised means with standard deviation [29, 35] and medians with ranges and IQR, respectively [31, 36]. Three papers divided ages into different categories [32, 33, 34]. The complete study characteristics can be found in Table 1. Overall, the studies included in our review represented a good geographical diversity with comparable initial T stages at presentation.

**TABLE 1:** Study characteristics of the included prediction models.

						Gender		AJCC TNM stage *n* (%)					Differentiation Degree *n* (%)			
Author, year	Study design	Enrolment period	Study setting	Study region	Age [median range (year)]/*n* (%)	Male, *n* (%)	Female, *n* (%)	T1	T2	T3	T4	Tis	Low	Moderate	Well	Undifferentiated
Zlobec et al. 2005 [26]	Prospective cohort	NI^a^	Cancer Hospital	Montreal, Canada	NI^a^	NI^a^	NI^a^	NI^a^	NI^a^	NI^a^	NI^a^	NI^a^	NI^a^	NI^a^	NI^a^	NI^a^
Zhou et al. 2022 (OS) [25]	Prospective cohort	2020–2021	Cancer Hospital	Fuijan, China	NI^a^	NI^a^	NI^a^	NI^a^	NI^a^	NI^a^	NI^a^	NI^a^	NI^a^	NI^a^	NI^a^	NI^a^
Zhao et al. 2023 [27]	Existing registry	NI^a^	NI^a^	NI	NI^a^	NI^a^	NI^a^	NI^a^	NI^a^	NI^a^	NI^a^	NI^a^	NI^a^	NI^a^	NI^a^	NI^a^
Xue et al. 2021 [28]	Existing registry	NI^a^	Cancer Hospital and Research Laboratory	Italy, USA, Japan	NI^a^	NI^a^	NI^a^	NI^a^	NI^a^	NI^a^	NI^a^	NI^a^	NI^a^	NI^a^	NI^a^	NI^a^
Wei et al. 2021 [29]	Existing registry	NI^a^	NI^a^	NI^a^	NI^a^	NI^a^	NI^a^	NI^a^	NI^a^	NI^a^	NI^a^	NI^a^	NI^a^	NI^a^	NI^a^	NI^a^
Sun et al. 2020 [30]	Retrospective cohort	2010–2016	Cancer Hospital	Fuzhou, China	54.9 ± 12.1^c^	36 (36.0)	64 (64)	NI^a^	35 (35)	65 (65)	NI^a^	NI^a^	NI^a^	NI^a^	NI^a^	NI^a^
Mbanu et al. 2022 [36]	Existing registry	2008–2019	Cancer Hospitals, clinics, radiotherapy	All over UK	66.5 (31–90)^d^	226 (70.2)	96 (29.8)	NI^a^	44 (13.7)	243 (75.5)	35 (10.9)	NI^a^	NI^a^	NI^a^	NI^a^	NI^a^
Lu et al. 2022 [31]	Retrospective cohort	2012–2021^b^	Cancer Hospital	Peking, China	61 (53.25, 69)^d^	158 (69.9)	68 (30.1)	NI^a^	11 (4.9)	175 (77.4)	40 (17.7)	NI^a^	26 (11.5)	182 (80.5)	18 (8.0)	NI^a^
Shin et al. 2022 [35]	Retrospective cohort	2000–2013	Cancer Hospital	Seoul, South Korea	55.5 ± 11^c^	742 (68.1)	347 (31.9)	5 (0.5)	128 (11.8)	798 (73.3)	158 (14.5)	NI^a^	36 (3.3)	766 (70.3)	222 (20.4)	Mucin 56 (5.1)
Wu et al. 2020 (ATV) [32]	Prospective cohort	2015–2017	Cancer Hospital	Hebei, China	≤ 60; 52 (65.8), > 60; 27 (34.2)	46 (58.2)	33 (41.8)	NI^a^	11 (13.9)	54 (68.4)	14 (17.7)	NI^a^	Non‐mucinous 69 (87.3), mucinous 10 (12.7)	NI^a^	NI^a^	NI^a^
Jiang et al. 2021 [33]	Retrospective cohort	2010–2018	Cancer Hospital	Guangdong, Fujian, China	< 65; 351 (82.0), ≥ 65; 77 (18.0)	296 (69.2)	132 (30.8)	NI^a^	NI^a^	199 (46.5)	229 (53.5)	NI^a^	332 (77.6)	NI^a^	96 (22.4)	NI^a^
Liu et al. 2021 (OS) [34]	Existing registry	2004–2016	Cancer Hospital	All over the US	< 60; 1484 (41.2), ≥ 60; 2115 (58.8)	2275 (63.2)	1324 (36.8)	Stage I 236 (6.6), II 2221 (61.7), III 393 (10.9), IV 21 (0.6), NA 728 (20.2)					NI^a^	NI^a^	NI^a^ v	NI^a^
Liu et al. 2021 (CSS) [34]	Existing registry	2004–2016	Cancer Hospital	All over the US	< 60; 1484 (41.2), ≥ 60; 2115 (58.8)	2275 (63.2)	1324 (36.8)	Stage I 236 (6.6), II 2221 (61.7), III 393 (10.9), IV 21 (0.6), NA 728 (20.2)					NI^a^	NI^a^	NI^a^	NI^a^
Wu et al. 2020 (without ATV) [32]	Prospective cohort	2015–2017	Cancer Hospital	Hebei, China	≤ 60; 52 (65.8), > 60; 27 (34.2)	46 (58.2)	33 (41.8)	NI^a^	11 (13.9)	54 (68.4)	14 (17.7)	NI^a^	Non‐mucinous 69 (87.3), mucinous 10 (12.7)	NI^a^	NI^a^	NI^a^
Zhou et al. 2022 (DFS) [25]	Prospective cohort	2020–2021	Cancer Hospital	Fuijan, China	NI^a^	NI^a^	NI^a^	NI^a^	NI^a^	NI^a^	NI^a^	NI^a^	NI^a^	NI^a^	NI^a^	NI^a^

^a^ No information.

^b^ January 2012–June 2021.

^c^ [Mean (SD)].

^d^ [Median (IQR or range)].

### 3.2 Radiotherapy Dose‐Schedule Relationships

There is currently a lack of consensus about the optimum radiation schedule for treating rectal cancer in terms of fractionation, and this was reflected in our review. Eight papers reported using doses between 45 and 50.4 Gy in 25 to 28 fractions [27, 29, 30, 31, 32, 33, 34, 35], while two papers reported on treatment involving 50 Gy in 25 fractions [26, 27]; one paper utilised 45Gy in 25 fractions [36]. However, one paper, Zlobec et al. [26] utilised high‐dose‐rate (HDR) brachytherapy and was subsequently excluded from our meta‐analysis.

According to the Radiation Dose Fractionation Third Edition, there is Grade A evidence supporting the use of either 45 or 50 Gy, and Grade C evidence for an optional boost of 5.4 Gy in 3 fractions. Similar rates of pathological complete response (pCR) or 2‐year disease‐free survival (DFS) are observed between these parameters [39].

In total, six papers did not exclusively focus on radiotherapy (RT), and four [40, 41, 42, 43] of these had the administration of RT (whether given or not) as a predictive factor in the final model. This introduced a certain degree of ambiguity, as there are arguments both in favour of the inclusion and exclusion of these four papers. Given that RT was not universally administered to all patients, the model is technically not exclusively an RT‐specific model. On the other hand, considering the administration of RT as a factor in the model attempts to address this issue. In the end, to maintain consistency across samples, papers that reported the administration of radiotherapy as a predictive factor were excluded from this review.

Capecitabine or 5‐fluorouracil (5‐FU) was the chemotherapy drug commonly used concurrently with radiotherapy for the treatment of recruited patients. In our review, we were able to compare the results from the treatment of rectal cancer patients utilising these two drugs as both agents have demonstrated comparable tumour downstaging rates [42]. However, one paper, Zlobec et al. [26] utilised high‐dose‐rate (HDR) brachytherapy and was subsequently excluded from our meta‐analysis.

Consequently, these papers [40, 41, 42, 43, 44, 45, 46] were not integrated into the review, acknowledging the difficulties in directly comparing them with other models in the context of predicting radiotherapy response.

### 3.3 Clinical Outcomes

The clinical outcomes predicted in the reviewed studies were broadly divided into two categories. The first category focused on histopathological response, specifically pathological complete response (pCR) or Tumour Regression Grade (TRG). The criteria for defining pCR included AJCC criteria [38] or Union for International Cancer Control (UICC) criteria [47]. A complete response was indicated by y[T0N0] response. However, a greater number of criteria were used in reporting TRG, with most studies referencing AJCC, Dworak system [48], Mandard criteria [49] and RCRG criteria [50]. Additionally, one paper utilised a pre‐specified clinical criteria defined by Habr‐Gama et al. [51].

The second category involved survival analysis. In most papers, the overall survival was defined as the duration from diagnosis to the most recent follow‐up date or date of death [25, 28, 35]. However, one study used the date from surgery as the starting point [29]. The follow‐up assessments for measuring response to treatment varied significantly; based on imaging alone versus imaging and clinical examination versus imaging, examination and serum CEA levels. Despite differences in modalities, the timing of the follow‐ups was consistent throughout the follow‐up period between different articles.

### 3.4 Sample Size

In total, 8343 patients were recruited in all the studies that were included. The reported sample sizes ranged from 42 to 4038 patients, with a median of 229. The outcome of interest (event) was defined as rates of response based on survival or histopathology. The number of events varied from 10 to 1047, with a median of 57.5. The lower end of this range was mostly contributed to by models based on novel genetic or protein markers. Studies that recruited patients from pre‐existing cancer registries accounted for the upper limit of this range. Half of the studies [25, 26, 27, 30, 31, 32] had fewer than 10 events per variable (EPV). A low EPV suggests restricted generalisability of a model to independent cohorts, as the predictive performance of these models is expected to diminish when applied to independent samples [52].

### 3.5 Modelling Method

The primary modelling methods employed were Cox proportional hazards regression (*n* = 5, 41.67%) [25, 27, 29, 34, 35] and logistic regression (*n* = 5, 41.67%) [30, 31, 32, 33, 36], with additional utilisation of machine learning techniques (*n* = 2, 16.67%) [26, 28]. Regarding the prediction horizon, the durations varied from 1 to 7 years. The majority of studies concentrated on predicting survival outcomes for a 1–3‐year horizon (*n* = 3, 40.0%) [25, 28, 35] while one study extended the prediction period to cover 1–7 years [29].

### 3.6 Model Performance

11 out of 12 papers reported at least one measure of calibration or discrimination. Calibration was evaluated in 8 models (67%), predominantly using calibration plot (*n* = 6) and Hosmer–Lemeshow test (*n* = 2). Discrimination metrics were more diverse, with C‐statistic (*n* = 10) [25, 28, 29, 30, 31, 32, 33, 34, 35, 36], Log rank (*n* = 1) [25] and risk group (*n* = 2) [25, 34], all being employed.

### 3.7 Model Presentation

Most papers (*n* = 9) [27, 28, 29, 30, 31, 32, 33, 34, 36] were presented in the form of nomograms. One model utilised a sum score [25] and another used a forest‐based classification tree [26]. None of the nomograms or models reported on the intercept or baseline hazard as required for external validation [53, 54]. It is challenging to calibrate a survival model to new populations without adjusting for baseline differences in predictive variables (using the intercept) and accounting for the underlying risk of an event independent of patient‐specific factors (through baseline hazard).

### 3.8 Validation of Selected Models

Most of the developed models underwent internal validation, employing methods such as random and non‐random dataset splits (*n* = 4) [25, 28, 31, 35], bootstrapping (*n* = 3) [29, 30, 33] or cross validation (*n* = 1) [26]. However, three papers used assessment of apparent performance of the model, which is not considered a true internal validation method [27, 32, 36]. Furthermore, only two models had undergone external validation. Among the two externally validated models, the study by Xue et al. [28] utilised datasets from different geographic regions, while Zhou et al.'s study [25] employed datasets from different time periods. Details about model characteristics are detailed in Table 2.

**TABLE 2:** Model characteristics of the included prediction models.

Author, year	Modelling method	Sample size	Events *n* (%)	Candidate	Final	EPV or EPP	Selection of candidate predictors	Selection of final predictors	Internal validation	External validation	Calibration	Discrimination	Overall performance
Zlobec et al. 2005 [26]	Machine learning techniques^a^	62	19 (30.6)	5	3	3.8	Based on prior knowledge	Random forest method	Cross validation	None	Not evaluated	Not evaluated	Not evaluated
Zhou et al. 2022 [25]	Cox regression	42	10 (13.9)	17	5	0.6	Based on prior knowledge	LASSO selection	Non‐random split data	Temporal	Not evaluated	AUC graph/log‐rank test/risk group curves	Not evaluated
Zhao et al. 2023 [27]	Cox regression	46	24 (52.2)	9	9	2.4	Based on univariable associations	Other^b^	None (apparent performance)	None	Calibration plot	Not evaluated	Not evaluated
Xue et al. 2021 [28]	Machine learning techniques (survival methods)	232	70^c^	4	4	17.5	Based on prior knowledge	Random forest method	Non random split data	Geographical	Not evaluated	AUC graph	Sensitivity 83.33% (10/12), specificity 61.90% (13/21), accuracy 69.70% (95% CI, 64.53%–74.87%)
Wei et al. 2021 [29]	Cox regression	293	139 (47.4)	14	6	9.9	Other	LASSO selection	Bootstrap, Ext	None	Calibration Plot	C‐statistic/AUC graph	Not evaluated
Sun et al. 2020 [30]	Logistic regression	100	32 (32.0)	15	4	2.1	Based on univariable associations	Pre‐specified model (not selection)	Bootstrap	None	Calibration Plot	C‐statistic/AUC graph	Not evaluated
Mbanu et al. 2022 [36]	Logistic regression	322	161 (50.0)	18	9	8.9	All available predictors	Pre‐specified model (not selection)	None (apparent performance)	None	Calibration Plot	C‐statistic/AUC graph	Not evaluated
Lu et al. 2022 [31]	Logistic regression	226	45 (19.9)	20	5	2.3	All available predictors	Backward elimination	Non random split data	None	HL test	AUC graph	Not evaluated
Shin et al. 2022 [35]	Cox regression	1089	198 (18.2)	17	3	11.6	All available predictors	Stepwise selection	Random split data	None	Not evaluated	AUC graph	Not evaluated
Wu et al. 2020 (ATV) [32]	Logistic regression	79	24 (30.4)	15	3	1.6	Based on prior knowledge	Unclear	None (apparent performance)	None	HL test	AUC graph	TPR: 95.8%
													TNR: 70.9%, PPV: 58.96%
													NPV: 97.84%
Jiang et al. 2021 [33]	Logistic regression	428	185 (43.2)	8	4	23.1	Based on univariable associations	Backward elimination	Bootstrap	None	Calibration Plot/HL test	AUC graph	Not Evaluated
Liu et al. 2021 (OS) [34]	Cox regression	4038	1074 (26.6)	15	10	71.6	Based on univariable associations	Other	Other	None	Calibration Plot	C‐statistic/risk group curves	Not Evaluated
Liu et al. 2021 (CSS) [34]	Cox regression	4038	NI	15	9	NI	Based on univariable associations	Other^d^	Other	None	Calibration Plot	C‐statistic/risk group curves	Not Evaluated
Wu et al. 2020 (without ATV) [32]	Logistic regression	79	24 (30.4)	15	2	1.6	Based on prior knowledge	Unclear	None (apparent performance)	None	HL test	AUC graph	TPR: 95.8%, TNR: 70.9%, PPV:58.96%, NPV: 97.84%
Zhou et al. 2022 (DFS) [25]	Cox regression	72	10 (13.9)	34	5	0.3	Based on prior knowledge	LASSO selection	Non‐random split data	Temporal	Not evaluated	AUC graph/log‐rank test/risk group curves	Not Evaluated

^a^ Random forest model.

^b^ Primarily describes the results of univariate analysis and logistic regression without specifying the variable selection method.

^c^ 70 patients in the training and testing group; no information is provided on the other cohorts.

^d^ Included a multi‐step process including the Kaplan–Meier method, log‐rank test, competitive risk analysis, cumulative incidence function (CIF) calculation, CIF curves, Grey's test, and finally, a multivariate analysis through a Cox regression model.

### 3.9 Predictive Factors

The addition of novel treatment markers or expression of genes/proteins to clinical factors is used to increase the predictive power of a prognostic model. In our review, the expression of novel genes was incorporated into six papers, and pre‐treatment biomarkers were included in five individual papers. Interestingly, other than pre‐treatment Neutrophil‐to‐lymphocyte Ratio (NLR), none of the pre‐treatment factors were utilised in more than one publication. This is shown in Table 3. The expression of 17 genes and proteins were utilised in the included papers. Six factors were utilised in two papers, and the rest only appeared in one paper. This is represented in Table 4.

**TABLE 3:** Frequency of treatment response biomarkers as predictive factors.

Treatment response biomarkers	Frequency
Pre‐treatment neutrophil‐to‐lymphocyte ratio (NLR)	2
Pre‐treatment platelet to lymphocyte ratio (PLR)	1
Pre‐treatment Prognostic Nutritional Index (PNI)_	1
Lymphocyte	1
Pre‐treatment haemoglobin	1
Serum alkaline phosphate (ALP)	1
Serum albumin	1
Lymphocyte to monocyte ratio (LMR)	1
Sodium to globulin ratio (SGR)	1
Collagen feature support factor machine classifier (CFS‐SVM)	1
Fibrinogen‐Albumin Ratio Index (FARI)	1

**TABLE 4:** Frequency of gene/protein expression as predictive factors.

Gene/proteins expression	Frequency
Cytochrome P450 1b1 (CYP1B1)	2
Dopa decarboxylase (DDC) gene	2
Anoctamin‐1 (ANOI) expression	2
Right open reading frame kinase 3 (RIOK3)	2
Apoptosis‐associated protein kinase‐like 1 (DAPL1)	2
Tumour stem marker CD44V6	2
Asparagine‐linked alpha‐1,2‐glucosyltransferase (ALG10)	1
Growth hormone receptor (GHR)	1
Heat shock protein family A (Hsp70) member 2 (HSPA2)	1
Fms‐like tyrosine kinase 3 (FLT3)	1
Angiopoietin‐1 (ANGPT)	1
Zinc finger protein 337 (ZNF337)	1
Pleiomorphic adenoma gene‐like 2 (PLAGL2)	1
Vascular endothelial growth factor (VEGF)	1
B‐Cell lymphoma 2 (BCL‐2)	1
Cyclin‐dependent kinase inhibitor 1 (CDKN1a, p21)	1
41 Gene pair signature (41‐GPS)	1

## 4 PROBAST

**FIGURE 4:**
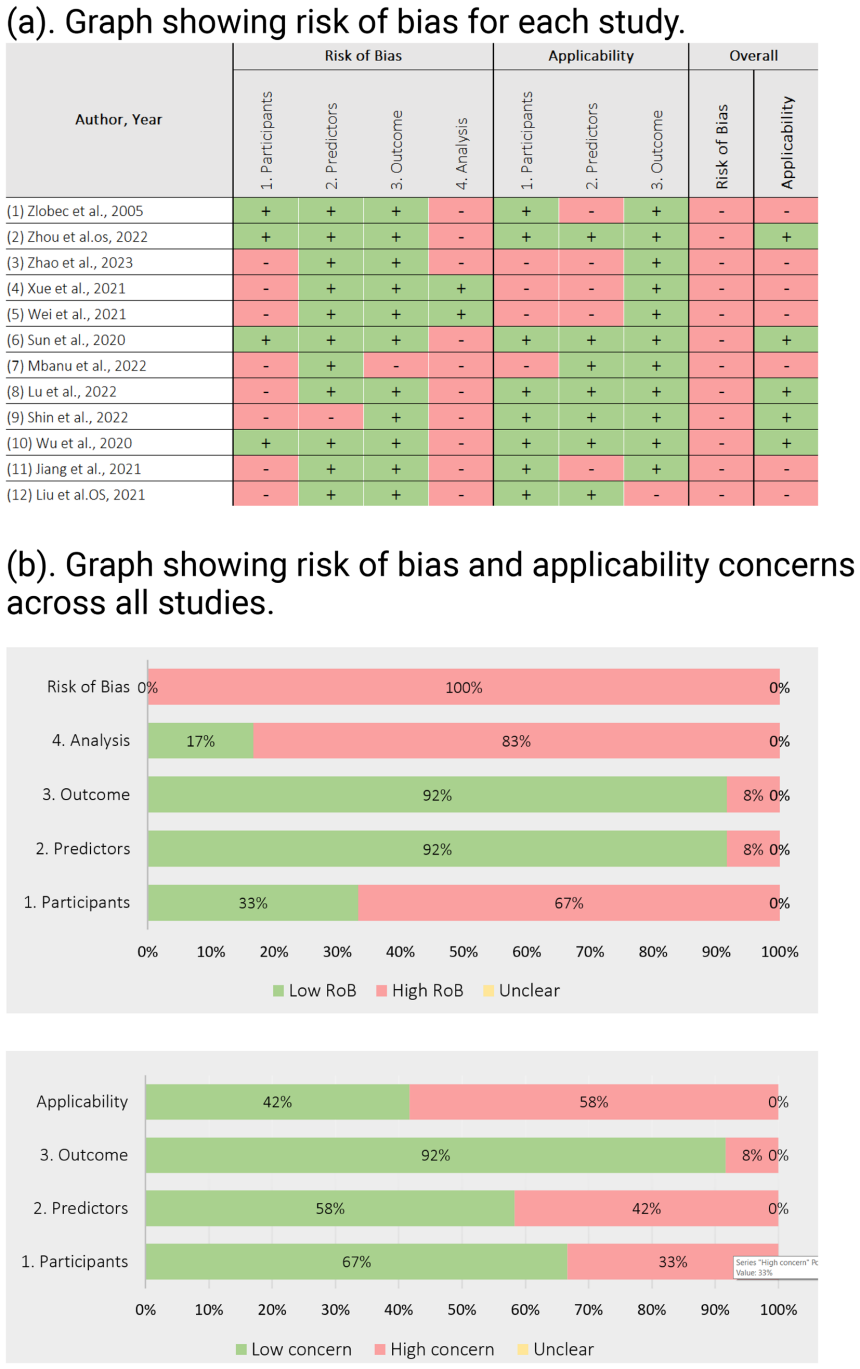
(a) Graph showing the risk of bias for each study. (b) Graph showing risk of bias and applicability concerns across all studies.

Each of the included papers demonstrated a high risk of bias in at least one of these domains, resulting in an overall high risk of bias. Notably, participant selection and analysis methods were the most frequently cited reasons for a high risk of bias in 67% and 89% of all papers, respectively. The results of the risk of bias assessment are diagrammatically depicted in Figure 4. A similar approach was utilised for the evaluation of applicability of the included models.

### 4.1 PROBAST (A) Risk of Bias

There are four domains used to assess the risk of bias in the included studies: Participants, predictors, outcome and analysis.

As illustrated in Figure 4, the risk of bias was minimal (< 10%) regarding the predictors utilised and the outcomes predicted. Studies benefitted from the use of standardised definitions for defining the predictors analysed (such as AJCC criteria for TNM staging). The time periods at which the variables were analysed were also consistent across studies. Similarly, a uniform definition was employed in assessing outcomes such as tumour regression grade or time to event (e.g., death).

In contrast, a high risk of bias was observed in the analysis component of these models. Most of the developed models had not undergone external validation. Another issue was the improper handling of continuous predictors during the analysis. Furthermore, the retrospective data collection was also a contributing factor. The implications of these findings will be discussed in the discussion section.

### 4.2 PROBAST (B) Applicability

More than half of the studies included in this review (58%) had a high risk of bias regarding the applicability of the model in independent patient cohorts. One‐third of these studies had significant concerns regarding the methods used to recruit patients during the model's development. Utilising single‐centre cohorts may fail to fully capture the heterogeneity present in the general population of rectal cancer patients worldwide. For example, the national bowel cancer screening programs in the United Kingdom are designed to detect asymptomatic cancers at an early stage compared to countries (such as China) that do not have such programmes. This can lead to potentially significant differences between patient cohorts from different countries.

Furthermore, less than half of the studies (42%) raised concerns regarding the predictors they utilised. This issue was more pronounced in studies focusing on proteomics, genomics or the discovery of unique biomarkers during model development. Since these novel biomarkers are not routinely collected, the applicability of such models is subject to the availability of these unique markers in routine clinical practice.

Only 8% of the studies raised significant concerns regarding applicability based on their predicted outcomes.

## 5 Discussion

This review identifies, summarises and scrutinises currently published prediction models for radiotherapy in rectal cancers. This will help provide context for comparing different models and evaluating their potential for adoption into clinical practice.

The CHARMS checklist streamlines the process of data collection for all variables needed to evaluate the risk of bias using the PROBAST tool. All included papers had a high risk of bias in at least one of these domains, leading to a high overall risk of bias. The most common causes of a high risk of bias were participants and analysis.

Recruitment of study participants is the first and the most important step in the development of any prediction model. It is important to identify and address any potential bias present in the raw data used to develop the model. Otherwise, the same bias might affect the predictions made by the model.

One possible solution is to collect a large sample size by utilising pre‐existing registries (e.g., SEER database). However, this method does not fully mitigate inherent selection bias. This is a particularly significant concern if there are multiple contributing centres. Furthermore, another factor to consider is the risk of attrition bias, especially in the cohort of non‐responders. Indeed, a complete case analysis can itself accentuate the exclusion bias for poor responders and those lost to follow‐up.

The same argument also applies to retrospective data collection methods. As an example, serum Carcinoembryonic Antigen (CEA) concentrations are not always routinely collected. The availability of this information could be influenced by specific clinical conditions or events. This irregularity in data collection for CEA levels represents possible confounding (i.e., patients being recruited into other studies).

One way to overcome these limitations is through external validation. According to the PROBAST definition, cohorts utilised for external validation should differ based on geographical, investigator or temporal (time) patient populations [21]. In this review, only two studies had true external validation. The remaining papers randomly allocated a 70:30 training: validation split from the same population. In cases where the two cohorts were balanced and comparable, it can be argued that these biases carried over to the external validation cohort.

Similarly, in handling predictors, converting a continuous variable, such as age or CEA level, to a categorical variable results in a loss of predictive power [55]. This risk is more pronounced when cut‐offs are not determined through a receiver operating characteristic curve.

A high risk of bias in applicability was reported for most studies included in our review (58%). A third of the studies had a high risk of bias regarding participants or predictors. This was mainly due to the inclusion of studies that focused on discovering unique biomarkers, as well as studies that utilised genomics or proteomics during model development. These novel biomarkers are not routinely collected in clinical practice. Therefore, the applicability of such models depends on the availability of these specific markers.

A commonly used rule of thumb in statistical modelling is to employ at least ten events per variable (EPV) analysed. This aims to ensure that there are an adequate number of cases (events) for each predictor variable. However, more sophisticated methods for sample size determination are now available. These include power analysis and resampling techniques. These techniques are designed to adjust for the complexity of the model and the specific goal of the analysis [52]. Nonetheless, the 10 EPV method remains a good rule of thumb. In our analysis, four papers had a lower than ten EPV. Additionally, these studies did not report the complete regression calculation utilised.

Another key metric that was missing from these publications was the rates of missing raw data in the collected sample. These papers also lacked sufficient information regarding the use of imputation techniques to address any missing variables (or the extent to which these techniques had been used). In particular, these studies lacked an explicit statement acknowledging or denying the use of imputation during model development. This meant that the rates of missing data could not be calculated from the study characteristics, as the ambiguity makes it difficult to assume complete case analysis was carried out during model development. Ultimately, this issue could directly impact the validity of predictions made by these models.

Another common hindrance in analysing these papers was the tendency to report on the predictive power of novel biomarkers or gene signatures, both individually and in combination with clinical factors. The addition of novel biomarkers to any prediction model will make the model's applicability entirely dependent on the biomarker's availability. Unfortunately, the included models did not report on the predictive ability of using the combination of clinical factors without the inclusion of the identified biomarker.

It is important to assess the baseline power of regularly collected clinical parameters. This analysis will allow for a better understanding of the independent contribution of these variables in predictive models.

The primary objective of the PROBAST models is to discern combinations of predictive factors, that is, prediction model rather than individual factors. Therefore, we separately recorded the frequency of the baseline demographic and clinical factors used in these models. Clinical and demographic factors were featured in 10 out of 15 models. Age was incorporated in five models, whereas grade and T stage of tumour were utilised in four models each. Moreover, M stage, gender and tumour size were featured in three models and CEA (Carcinoembryonic Antigen) in two models. This trend underscores the substantive importance of these clinical parameters in predicting outcome to treatment. The frequencies of commonly used predictive factors in the included models are summarised in Table 5.

**TABLE 5:** Frequency of variables used as predictive factors.

Variable	Frequency
Age	5
Grade	4
T stage	4
Gender	3
Stage	3
M stage	3
Tumour size	3
N stage	2
Carcinoembryonic antigen (CEA) levels	2
Site of metastasis^a^	2
Interval week^b^	1
Real tumour volume	1
Tumour surface area	1
Tumour compactness	1
Marital status	1
Insurance status	1
Grouped variables	
Genes/proteins	6
Treatment response biomarkers	5

^a^ Site of metastasis from primary rectal cancer: Bone, Brain, Liver, Lung.

^b^ Interval in number of weeks between the end of nCRt and surgery.

It is important to understand these findings in the context of previously published review articles in this field. A systematic review conducted in 2019 focused on prediction models that attempted to predict the response of colorectal cancer patients to surgery [56]. Similarly, a separate umbrella study of systematic reviews and d was conducted in 2020. This study concentrated on models predicting rates of local recurrence and metastases, regardless of the treatment modality [57]. In this study, a total of 24 models were identified. Ten models concentrated on predicting responses to curative resection, while six focused on predicted rates of recurrences or metastases regardless of the type of treatment administered. The remaining models focused on metastases at presentation with synchronous metastases, radiomics or were specific to chemotherapy.

From these previous systematic reviews, there were only three publications that fulfilled our inclusion criteria. All three models had already been identified by our search string in our initial search. However, due to the inclusion of post‐treatment factors (e.g., post treatment T stage), these models were excluded from our analysis.

This review has therefore identified clinically predictive models for radiotherapy in rectal cancer that can be used at baseline (i.e., before initiating treatment). Despite being statistically robust, these models have limited applicability in real‐world clinical practice. The future aim following this review is to inform the development of a prediction model for CXB in rectal cancer. However, in contrast to EBRT, contact X‐ray brachytherapy is used relatively less frequently. Currently, CXB is only practiced in a limited number of centres internationally. This makes the development of a model for CXB susceptible to challenges related to achieving an adequate sample size and EPV. The Clatterbridge Cancer Centre, UK, has the world's largest cohort of rectal cancer patients who have been treated with CXB. To ensure a sufficient sample size for developing a model for response to Papillon treatment, we plan to conduct targeted data retrieval from the comprehensive database at this institution.

Similarly, in order to tackle problems with the validation of future models, attempts will be made to contact other CXB treatment centres to provide geographically different cohorts. Lastly, we aim to preserve transparency by reporting this according to the Transparent reporting of a multivariable prediction model for individual prognosis or diagnosis (TRIPOD): the TRIPOD Statement [53].

Further recommendations based on the issues highlighted in this review include informing sample size calculations using advanced statistical techniques designed to minimise the risk of overfitting and reduce the mean absolute error, as discussed by Riley et al. [52]. Additionally, the future model should prioritise clinical parameters, incorporating tumour‐specific factors such as T stage, N stage and tumour size, in conjunction with patient‐specific factors such as age and overall fitness level.

While this review exclusively evaluated single‐modality models (based on clinical parameters), future efforts should focus on integrating multiple modalities, such as MRI imaging, histopathology of biopsy samples and colonoscopy images. This is because models based on multimodality inputs have demonstrated superior predictive performance compared to single‐modality models [58].

In addition to collecting data from a cohort of patients treated with CXB to develop a CXB‐specific model, efforts should also prioritise creating and prospectively preserving a dedicated ‘watch‐and‐wait’ database for patients offered CXB therapy. Such a database would facilitate research into watch‐and‐wait approaches involving CXB, similar to the international watch‐and‐wait database established for external beam chemoradiotherapy in rectal cancer patients [59].

## 6 Conclusion

In conclusion, our study presents a comprehensive overview of the existing clinical prediction models for predicting response to neoadjuvant radiotherapy in rectal adenocarcinoma patients. Models predicting response based on TRG yielded an AUC of 0.82 (95% CI 0.74–0.89). Whereas, models predicting response using pCR demonstrated an AUC of 0.76 (95% CI 0.71–0.82). However, these models were found to have a high risk of bias in patient recruitment and low applicability for the general population. These limitations have important implications for the predictions generated by these models. The design of a future CXB‐specific prediction model should prioritise a dedicated data collection process. It should aim to recruit a pre‐calculated sample size to achieve an adequate EPV. Furthermore, the predictive factors identified in this review should be evaluated as potential predictors for the CXB‐specific model.

## Author Contributions

**Muneeb Ul Haq:** data curation (lead), formal analysis (lead), investigation (lead), software (lead), writing – original draft (lead), writing – review and editing (supporting). **D. Mark Pritchard:** data curation (supporting), formal analysis (supporting), funding acquisition (lead), supervision (lead), writing – review and editing (lead). **Arthur Sun Myint:** formal analysis (supporting), funding acquisition (equal), project administration (equal), supervision (lead), writing – review and editing (supporting). **Muhammad Ahsan Javed:** data curation (supporting), formal analysis (supporting), supervision (supporting), writing – review and editing (supporting). **Carrie A. Duckworth:** project administration (supporting), software (supporting), writing – original draft (supporting). **Ngu Wah Than:** data curation (supporting), formal analysis (supporting), methodology (equal). **Laura J. Bonnett:** methodology (equal), resources (supporting), visualization (supporting), writing – review and editing (supporting). **David M. Hughes:** formal analysis (supporting), investigation (supporting), methodology (equal), project administration (lead), software (lead).

## Ethics Statement

This article draws upon previously conducted studies and does not introduce any novel research involving human participants or animals by any of the authors. Therefore, it does not necessitate approval from an independent ethics committee.

## Consent

The authors have nothing to report.

## Conflicts of Interest

The authors declare no conflicts of interest.

## Supporting information


Table S1.

## Data Availability

All authors were granted full access to the data involved in this study and bear responsibility for ensuring its integrity and the accuracy of its analysis. The datasets generated and/or analysed during this study are accessible from the corresponding author upon request.
